# Carnosine alleviates oxidative stress to prevent cellular senescence by regulating Nrf2/HO-1 pathway: a promising anti-aging strategy for oral mucosa

**DOI:** 10.3389/fphar.2025.1559584

**Published:** 2025-04-10

**Authors:** Haoan He, Chao Lv, Yuhong Xie, Wei Li, Zihang Ling, Bin Cheng, Xiaoan Tao

**Affiliations:** ^1^ Guanghua School of Stomatology, Sun Yat-sen University, Guangzhou, Guangdong, China; ^2^ Hospital of Stomatology, Sun Yat-sen University, Guangzhou, Guangdong, China; ^3^ Guangdong Provincial Key Laboratory of Stomatology, Sun Yat-sen University, Guangzhou, Guangdong, China

**Keywords:** carnosine, senescence, aging, metabolomics, oral mucosa

## Abstract

**Introduction:** Aging is associated with significant metabolic alterations that contribute to cellular senescence and age-related functional decline. As individuals age, an increased prevalence of oral diseases and a gradual decline in oral functions are observed. However, the metabolic shifts underlying oral mucosal aging remain unexplored.

**Methods:** We initially conducted histological analyses on the tongues from young (4-week-old), adult (4-month-old) and old (20-month-old) C57BL/6 mice to identify age-related alterations in the tongue mucosa. Subsequently, metabolomics analysis was performed to characterize metabolic profiles of mouse tongues across these age groups and identify metabolic biomarkers of oral mucosal aging. Then we validate the anti-senescence effect of carnosine and investigate its underlying mechanisms using a tert-butyl hydroperoxide (tBHP)-induced cellular senescence model in vitro. Finally, metabolomics analyses of human saliva and blood were conducted to explore associations between carnosine levels and systemic aging.

**Results:** Compared to young and adult mice, we observed epithelial atrophy and an accumulation of senescent cells in the tongue mucosa of old mice. After that, we found significant differences in the metabolic profiles among the young, adult, and old mouse tongues. Carnosine was identified as a potential biomarker of oral mucosal aging, as its levels declined significantly with age. Consistently, carnosine synthase 1 (CARNS1) activity decreased, and carnosinase 2 (CNDP2) activity increased with age in the tongue mucosa. Furthermore, carnosine protected oral epithelial cells from tBHP-induced cellular senescence by reducing oxidative stress, mitigating DNA damage, and downregulating Nrf2/HO-1 pathway. In humans, salivary and blood carnosine levels also declined with age and were significantly associated with age-related diseases.

**Discussion:** Our findings reveal dynamic metabolic reprogramming during natural oral mucosal aging and highlight the dual role of carnosine as both an aging biomarker and a therapeutic target for combating age-related mucosal degeneration. These insights support the development of novel carnosine-based interventions to preserve oral mucosal function, prevent age-related oral diseases, and improve oral health in the aging population, thereby advancing healthy aging.

## 1 Introduction

The globe meets the challenges of an aging population. According to the World Population Prospects 2024, the global average life expectancy will reach 73.3 years in 2024. It is expected that by the end of the 2070s, the global population aged 65 and above will reach 2.2 billion, surpassing the number of people under the age of 18 (United Nations Department of Economic and Social Affairs, 2024). Aging is described as an inevitable time-dependent functional decline that affects all living organisms (López-Otín et al., 2023). Aging-related changes lead to declined functions and increased risks of diseases and mortality. Cellular senescence, a hallmark of aging, is described as prolonged and generally irreversible cell cycle arrest (Gorgoulis et al., 2019). Although cellular senescence is beneficial to embryonic development, host immunity, tumor suppression and wound healing, the aberrant accumulation of senescent cells has been identified as the primary cause of age-related diseases, including chronic obstructive pulmonary disease (COPD), neurodegenerative diseases, atherosclerosis and cancers (Di Micco et al., 2021). Recently, clearing senescent cells emerged as a promising therapy to extend lifespan, alleviate age-related functional decline and improve other health indicators (Chaib et al., 2022).

Aging significantly affects the oral cavity (Ib et al., 2016). A previous study showed significant accumulation of senescent cells in monkeys’ gingival epithelium with aging (Hu et al., 2024). A recent study showed senescent cells accumulated in periodontal ligament and alveolar bone with aging and exacerbated the chronic inflammation in periodontal tissue through secreting senescence-associated secretory phenotype (SASP) and interacting with bacteria (Chen et al., 2022). In pulp tissue, CD51^+^/PDGFR-α^+^ human dental pulp stromal cells (hDPSCs), which were promising seeding cells for regenerative medicine, decreased in chronological senescence. While the chronologically senescent hDPSCs showed impaired self-renewal and higher ossificatory differentiation (Yao et al., 2023). With aging, oral mucosa exhibits an increased incidence of multiple diseases, including infectious diseases, oral potentially malignant disorders (OPMDs) and oral squamous cell carcinoma (OSCC) (Ib et al., 2016). The incidence of OSCC rises dramatically after the age of 40–49 years and reaches a plateau around the age of 70–79 years (Hussein et al., 2017). Additionally, older age at diagnosis of OSCC is associated with lower overall survival (OS) (Zanoni et al., 2019). Furthermore, the aging oral mucosa is characterized by altered mucosal sensation, increased permeability, and delayed wound healing (Ib et al., 2016). However, the mechanism of oral mucosal aging and the treatment against age-related changes remain unclear.

Emerging evidence indicated that metabolic dysregulations contributed to aging (Hamrick and Stranahan, 2020). Senescent cells undergo extensive metabolic reprogramming to survive through avoiding apoptosis (Wiley and Campisi, 2021). Recent studies indicated that metabolic profiles of plasma in older individuals are significantly different from those in younger people (Tian et al., 2022). Furthermore, the metabolic profiles of healthy agers differ from those of rapid agers (Hamsanathan et al., 2024). Additionally, it seems plausible that metabolites can serve as biomarkers of aging and metabolomics shows great promise in assessing biological age (Rist et al., 2017; Robinson et al., 2020). However, most metabolomic analyses focus on the alterations of global plasma and other biofluids (Pietzner et al., 2021), yet biofluid metabolomics lacks the capacity to offer the detailed insights into the aberrant metabolism occurring in local disease pathogenesis that tissue metabolomic analysis can provide (Saoi and Britz-McKibbin, 2021). To date, no metabolomic analysis has been conducted on oral mucosal aging, and the mechanisms linking metabolic changes to oral mucosal aging remain unknown.

Carnosine is a dipeptide composed of β-alanine and L-histidine, with high levels in skeletal muscle, myocardium and brain, especially in the olfactory bulb (Boldyrev et al., 2013). It is synthesized by CARNS1 and degraded by carnosinase (CN) (Boldyrev et al., 2013). Carnosine has been proven to have many functions, including regulating excitation-contraction coupling, exhibiting antioxidant activity, chelating metal ions, inhibiting protein carbonylation and acting as a pH buffer (Boldyrev et al., 2013). In aging research, carnosine shows promising potential for treating age-related disorders (José et al., 2015), including pulmonary fibrosis, myocardial ischemia reperfusion injury and age-related cataract (Dubois and Bastawrous, 2017; Zhao et al., 2020; Park et al., 2022). Additionally, it protects against neurodegenerative diseases such as Alzheimer’s disease, dementia, and Parkinson’s disease by inhibiting amyloid-β (Aβ) aggregation and reducing neuroinflammation (Banerjee et al., 2021; Spaas et al., 2021). However, the specific role of carnosine in oral mucosal aging remains unexplored.

This study was designed to investigate metabolic alterations during aging in mouse tongues and to identify potential metabolic biomarkers and therapeutic agents to mitigate oral mucosal aging. For the first time, we observed epithelial atrophy and the accumulation of senescent cells in the tongue mucosa of aging mice. Through untargeted metabolomic analysis, we identified carnosine as a key biomarker of oral mucosal aging, showing a significant age-related decline in its level. Furthermore, we found that carnosine alleviated tBHP-induced cellular senescence by reducing oxidative stress, mitigating DNA damage, and downregulating the Nrf2/HO-1 pathway. Our work provided a comprehensive understanding of oral mucosal aging at the metabolic level and established carnosine as a promising therapeutic candidate for developing new strategies to prevent and treat aging-related oral mucosal diseases.

## 2 Materials and methods

### 2.1 Animals

All mice involved in this study were C57BL/6 acquired from the Laboratory Animal Center, Sun Yat-sen University. Mice were housed under specific pathogen-free circumstances with a 12-h light/dark cycle and free access to tap water and food. Experimental mice were divided into three age groups: young mice (YM, 4-week-old), adult mice (AM, 4-month-old), and old mice (OM, 20-month-old). Mice were sacrificed via cervical vertebra decoupling under isoflurane inhalation anesthesia, and tongue tissues were collected immediately and then stored at −80°C. All animal experiments were executed with the approval of the Institutional Animal Care and Use Committee of Sun Yat-sen University (SYSU-IACUC-2024-001928).

### 2.2 Metabolomics analysis

Sample processing and analyses were performed by Luming Biological Technology, Inc. (Shanghai, China). The brief analysis processes are as follows, and detailed steps and parameters are available upon request. We collected tongue tissue from six mice in each group. For liquid chromatography-mass spectrometry (LC-MS) analysis, a Dionex Ultimate 3000 RS UHPLC with a Q-Exactive quadrupole-Orbitrap mass spectrometer (Thermo Fisher Scientific, Waltham, MA, United States) was used, operating in both ESI positive and negative ion modes. An ACQUITY UPLC HSS T3 column (1.8 μm, 2.1 × 100 mm) was employed. For gas chromatography-mass spectrometry (GC-MS) analysis, derivatized samples were analyzed on an Agilent 7890B gas chromatography system coupled to an Agilent 5977B MSD system (Agilent Technologies Inc., CA, United States). An HP-5MS fused-silica capillary column (30 m × 0.25 mm × 0.25 μm, Agilent J & W Scientific, Folsom, CA, United States) was used for separation. QC samples were injected at regular intervals throughout the analytical run to determine repeatability.

The original GC-MS and LC-MS data were processed using Analysis Base File Converter, MS-DIAL, and Progenesis QI for format conversion, baseline filtering, peak identification, integration, retention time correction, peak alignment, and normalization. After normalization, redundancy removal and peak merging were performed to obtain the data matrix. The data matrix was imported into R for analyzing and visualizing. Differential metabolites between groups were identified using one-way ANOVA (*p* < 0.05) and selected based on VIP values >1.0. Metabolic pathway enrichment analysis was performed using MetaboAnalyst 4.0 and the KEGG database, applying a threshold of impact values >0.1 and −log(P) values >2.0. Correlation analysis between differential metabolites was conducted using Spearman’s rank correlation coefficient in MetaboAnalyst 4.0 (*p* < 0.05; *ρ* < −0.5 or ρ > 0.5) and visualized as chord diagrams with the R package “circlize.” And the classification was based on Human Metabolome Database (HMDB) classification. Receiver operating characteristic (ROC) curve analysis was also performed using MetaboAnalyst 4.0.

Moreover, we extracted the data of carnosine-related metabolites in human serum and saliva from MetaboLights (MTBLS265 and MTBLS2108) and performed ROC curve analysis using MetaboAnalyst 4.0. Data from the association matrix, including standardized regression coefficients (β-estimates) and nominal *p*-values, were extracted from the open-access web server (https://omicscience.org/apps/mwasdisease/). Regression coefficients and nominal *p*-values were plotted in a heatmap using R version 4.1.0.

### 2.3 Cell culture

The cell lines used in this study were human dermal keratinocyte (HaCaT) and human oral epithelial cell (HOEC), which were kindly provided by Professor Xianyue Ren and Professor Guiqing Liao (Guanghua School of Stomatology, Sun Yat-sen University), respectively. Both cell lines were grown in DMEM (Gibco, #11965092) containing 10% FBS (TransGenBiotech, FS401) and 100 IU/mL penicillin/streptomycin at 37°C in a 5% CO_2_ humidified incubator. HaCaT and HOEC were treated with tBHP (Sigma Aldrich, #458139) to induce cellular senescence. Cells were seeded and cultivated in complete culture medium until they reached 50% confluence, with or without carnosine (Sigma-Aldrich, C9625) and N-acetyl-L-cysteine (NAC, Sigma-Aldrich, A2835) supplementation. tBHP was administered at 250 μM for 2 hours and 200 μM for 6 hours to HaCaT and HOEC, respectively (Li et al., 2020). Then cells were washed three times with PBS (Gibco, #10010023) and cultured for another 2 days with or without carnosine and NAC supplementation.

### 2.4 Histological analysis

Tongue tissues were collected from mice, rinsed three times with PBS, fixed in 4% paraformaldehyde (PFA) for 24 h at 4°C, and dehydrated in a 30% sucrose in PBS solution for 24–48 h. The fixed tongues were embedded in paraffin, cut into 4-μm-thick sections using a rotary microtome (Leica, AUTOCUTE), and stained with hematoxylin and eosin (H&E). Histologic images were captured with a slide scanner (Leica, Aperio AT2) and analyzed using ImageScope 11.0 software. The thickness of the tongue epithelium, defined as the total thickness of the stratum granulosum, stratum spinosum, and stratum basale (Wakamori et al., 2022), was measured at 15 random points per whole tongue.

### 2.5 Immunohistochemistry (IHC)

Paraffin-embedded tissues were dewaxed with Histoclear (Solarbio, YA0031) and rehydrated with a gradient of ethanol. Antigen was retrieved by incubating the sections for 5–10 min in 0.01 M citrate buffer (pH = 6.0). The slides were immersed in 0.1% Triton X-100 for 15 min, then blocked with normal goat serum (BOSTER, AR0009) for 60 min at room temperature. Sections were then incubated with primary antibodies at 4°C overnight and then were washed with PBS three times. The primary antibodies used in this experiment are shown in Table 1. Afterward, sections were incubated in 3% H_2_O_2_ for 30 min to block endogenous peroxidase activity. Secondary antibodies were incubated on slides at room temperature for 60 min. Tissue sections were stained with diaminobenzidine (DAB, Servicebio, G1212) and counterstained with hematoxylin. Images were captured using a slide scanner (Leica, Aperio AT2) and analyzed by ImageScope 11.0. The Ki-67 or p21^Waf1^ positive cells were counted manually and averaged in six high-power fields (HPFs, 400×). The expression levels of CARNS1 and CNDP2 were measured by H-score plugins in ImageScope 11.0.

**TABLE 1:** Primary antibodies used in the study.

Name	Company and Cat no.	Application
Anti-p21^Waf1^	Santa Cruz, sc-6246	1:200 (IHC), 1:500 (WB)
Anti-Ki-67	Abcam, ab16667	1:1,000 (IHC)
Anti-CARNS1	Abbexa, abx129855	1:50 (IHC)
Anti-CNDP2	Proteintech, 14925-1-AP	1:1,000 (IHC)
Anti-γH2A.X	Servicebio, GB11365-100	1:100 (IF), 1:500 (WB)
Anti-Nrf2	Santa Cruz, sc-365949	1:500 (WB)
Anti-HO-1	Abcam, ab189491	1:2000 (WB)
Anti-β-actin	Proteintech, 66009-1-Ig	1:5,000 (WB)
Anti-8-OHdG	Santa Cruz, sc-66036	1:300 (IF)

### 2.6 Immunofluorescence (IF)

Paraffin-embedded tissue sections were dewaxed, rehydrated, and antigen-retrieved as previously described in our IHC protocol. The slides were immersed in 0.3% Triton X-100 for 10–15 min, then blocked with 5% BSA for 60 min at room temperature. Slides were gently washed with PBS, followed by incubating with the primary antibody at 4°C overnight. Following primary antibody removal, the slides were incubated with the appropriate secondary antibodies for 1 h at room temperature in the dark. Nuclei were stained with DAPI (Beyotime, C1002) for 5 min. After washing, the sections were mounted with anti-fade mounting medium (Servicebio, G1401). Images were captured using a ZEISS Axio microscope and an Olympus FV3000 confocal microscope. Fluorescent signal intensity was analyzed using ImageJ. The primary antibodies used in this experiment were shown in Table 1. The fluorescent intensity was measured by ImageJ.

### 2.7 CCK-8 viability assay

HaCaT and HOEC cells were seeded in 96-well plates at a density of 1 × 10^4^ cells/cm^2^ and cultured in DMEM medium with gradient concentrations of carnosine (0, 2, 5, 10, 20 mM). After 2 days, the medium was replaced with 200 μL of fresh medium containing 20 μL of CCK-8 reagent (DOJINDO). Following a 2-h incubation, the absorbance at 450 nm was measured using a spectrophotometric microplate reader (BioTek Synergy H1).

### 2.8 Western blot

Total protein was extracted by RIPA lysis buffer (CWBIO, CW2333S), whose concentrations were quantified by BCA protein assay kit (CWBIO, CW0014S). Proteins were separated by SDS-PAGE and transferred to PVDF membranes. The membranes were blocked with 5% milk for 1 h at room temperature. Then the membranes were incubated with primary antibodies overnight at 4°C. Following washing with TBST, the membranes were incubated with appropriate secondary antibodies. Detection of labeled proteins was carried out using Immobilon Western Chemiluminescent HRP Substrate (Millipore, WBKLS0050). The antibodies utilized in this experiment were detailed in Table 1.

### 2.9 RNA isolation and quantitative real-time PCR (qRT-PCR)

Total RNA was isolated and purified from cell samples using the Easy Pure Fast Cell RNA Kit (Transgene, ER111), and the concentrations of RNA were measured by NanoDrop (Thermo). DNase treatment and reverse transcription were performed using the HiScript III All-in-one RT SuperMix (Vazyme, R333). The obtained templates were used for qRT-PCR with SYBR MasterMix (Vazyme, Q511) on an ABI Q5 (Thermo). All samples were normalized to β-actin. Primer sequences for qRT-PCR are shown in Table 2.

**TABLE 2:** Primer sequences for qRT-PCR.

Homo gene	Primer sequences (5′ > 3′)	
IL-1β	Forward	TGT​ACC​TGT​CCT​GCG​TGT​TG
	Reverse	ACG​GGC​ATG​TTT​TCT​GCT​TG
IL-6	Forward	CAA​TGA​GGA​GAC​TTG​CCT​GGT
	Reverse	GCA​GGA​ACT​GGA​TCA​GGA​CT
IL-8	Forward	ACA​CTG​CGC​CAA​CAC​AGA​AAT​TA
	Reverse	TTT​GCT​TGA​AGT​TTC​ACT​GGC​ATC
IL-18	Forward	TCT​TCA​TTG​ACC​AAG​GAA​ATC​GG
	Reverse	TCC​GGG​GTG​CAT​TAT​CTC​TAC
TNF-α	Forward	TAT​CCT​GGG​GGA​CCC​AAT​GT
	Reverse	AAA​AGA​AGG​CAC​AGA​GGC​CA
Nrf2	Forward	GTG​TGG​CAT​CAC​CAG​AAC​AC
	Reverse	GAC​ACT​TCC​AGG​GGC​ACT​AT
HO-1	Forward	AGT​CTT​CGC​CCC​TGT​CTA​CT
	Reverse	CTT​CAC​ATA​GCG​CTG​CAT​GG
NQO1	Forward	AAC​ACT​GCC​CTC​TTG​TGG​TG
	Reverse	GCT​CGG​TCC​AAT​CCC​TTC​AT

### 2.10 Senescence-associated β-Galactosidase (SA-β-Gal) staining

As previously described, SA-β-Gal staining was carried out on human cells at pH 6.0 and on mouse tissue at pH 5.5 (Amor et al., 2020). To stain the tongue with SA-β-Gal, the tissue was embedded in OCT, sectioned at a thickness of 12 μm, and stored at −20°C. After rehydration in PBS, staining was performed using the SA-β-Gal Staining Kit (CST, #9860) with a 15-min fixation followed by incubating in the staining solution at 37°C without CO_2_ for 96 h. Tissue sections were counterstained with 0.1% Nuclear Fast Red Solution (Beytime, G1320). Adherent cells were fixed for 10 min before being immersed in the SA-β-Gal staining solution at 37°C without CO_2_ for 48 h. Cells with cytoplasmic staining were scored as positive. Five high-power fields per well were counted and averaged to quantify the percentage of SA-β-Gal positive cells.

### 2.11 Determination of ROS

The intracellular ROS level was measured using the Reactive Oxygen Species Assay Kit (Beyotime, S0064S). Cells were collected 48 h after treatment with tBHP, carnosine and NAC. Afterward, cells were incubated with 10 μM Dihydroethidium (DHE) in the dark at 37°C for 20 min. Following washes with PBS, the mean fluorescence intensity (MFI) was then detected using flow cytometry.

### 2.12 Statistical analysis

Statistical analysis was performed using SPSS version 22.0. Statistical significance was determined using one-way ANOVA with Bonferroni correction. *P* value <0.05 was considered to indicate statistical significance. Results are illustrated using GraphPad Prism 10. The error bars indicate the standard error of the mean (SEM).

## 3 Results

### 3.1 Epithelial atrophy and the accumulation of senescent cells in the tongue mucosa of elderly mice

**FIGURE 1:**
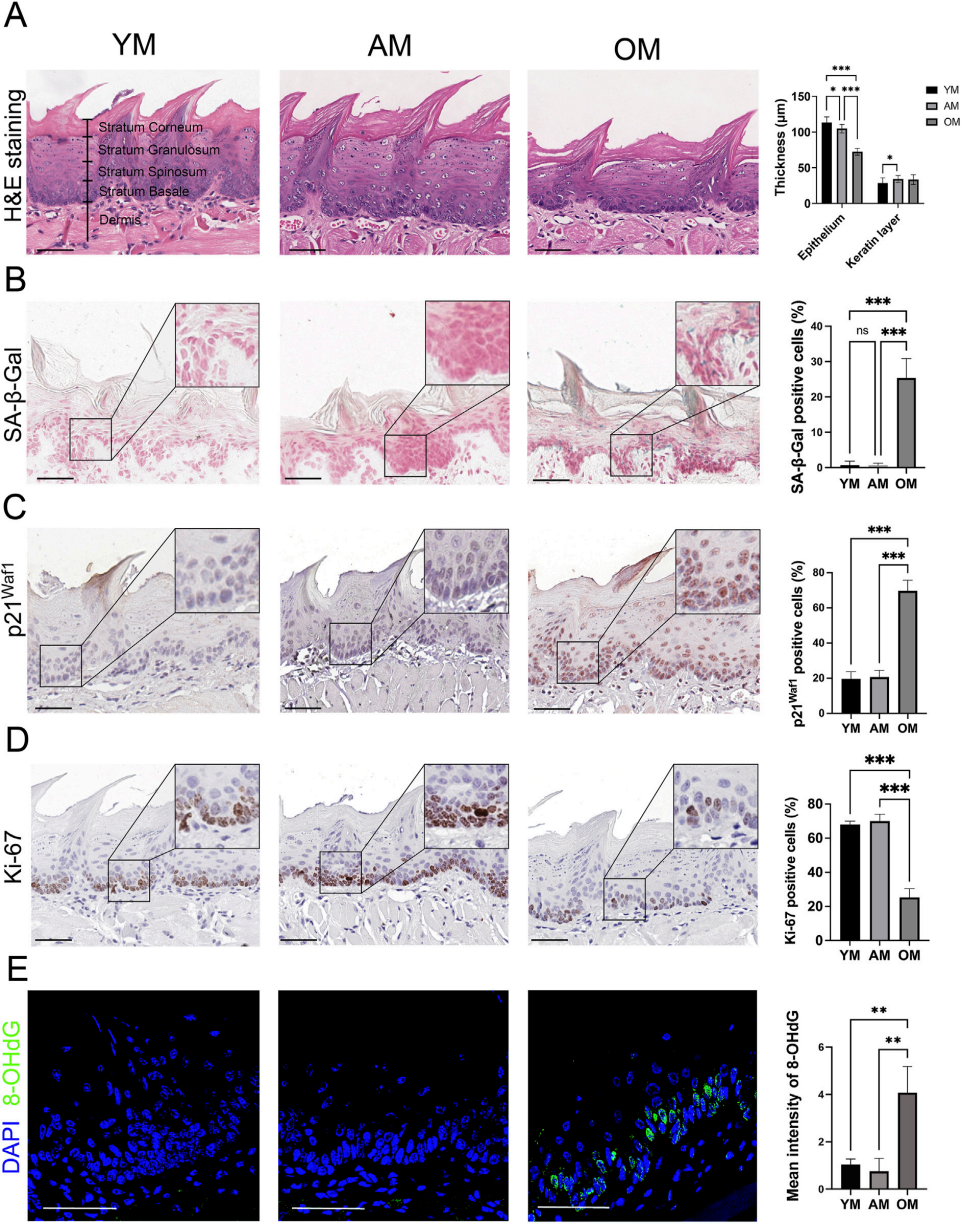
Epithelial atrophy and accumulation of senescent cells in the tongue mucosa of old mice. **(A)** Representative H&E images and quantification of epithelial thickness and keratin layer thickness in the tongue from young, adult and old mice. **(B)** Representative images of the dorsal surface of the tongue epithelium from YM, AM and OM assayed for SA-β-Gal activity are shown. In addition, quantification of the proportion of SA-β-Gal positive cells in YM, AM and OM is provided. **(C,D)** Representative images of immunohistochemical staining of **(C)** p21^Waf1^ and **(D)** Ki-67 in tongue epithelium of YM, AM and OM are shown. Quantification of the proportion of positive cells in YM, AM and OM is provided. **(E)** Representative images and quantification of immunofluorescence of 8-OHdG in the tongue epithelium from YM, AM and OM are shown. For all relevant figures, scale bars: 50 μm. Data are represented as mean ± SEM (*p < 0.05, **p < 0.01 and ***p < 0.001).

H&E staining revealed significant epithelial atrophy in the tongue mucosa of OM, with a notable reduction in epithelial thickness compared to YM and AM. However, there is no significant reduction in the thickness of the keratin layer with aging (Figure 1A). Then we further detected cellular senescence with SA-β-Gal staining and immunohistochemical staining of p21^Waf1^. Few SA-β-Gal positive cells were observed in the tongue mucosa of YM and AM, whereas the SA-β-Gal positive epithelial cells significantly increased in the tongue mucosa of OM (Figure 1B). A significant increase of p21^Waf1^ positive cells was observed in the epithelial cells of tongue mucosa of OM compared with YM and AM, with no significant difference between YM and AM (Figure 1C). Additionally, the proportion of Ki-67 positive cells in tongue epithelium declined significantly in OM compared with YM and AM (Figure 1D). DNA damage in the tongue mucosa was assessed by measuring 8-OHdG levels, which were significantly elevated in the tongue epithelium of OM compared to YM and AM (Figure 1E). These results suggest that epithelial atrophy, accumulation of senescent cells, and increased DNA damage occur in the tongue mucosa of OM, but not in YM and AM.

### 3.2 Metabolic alterations in tongue mucosal aging in mice

**FIGURE 2:**
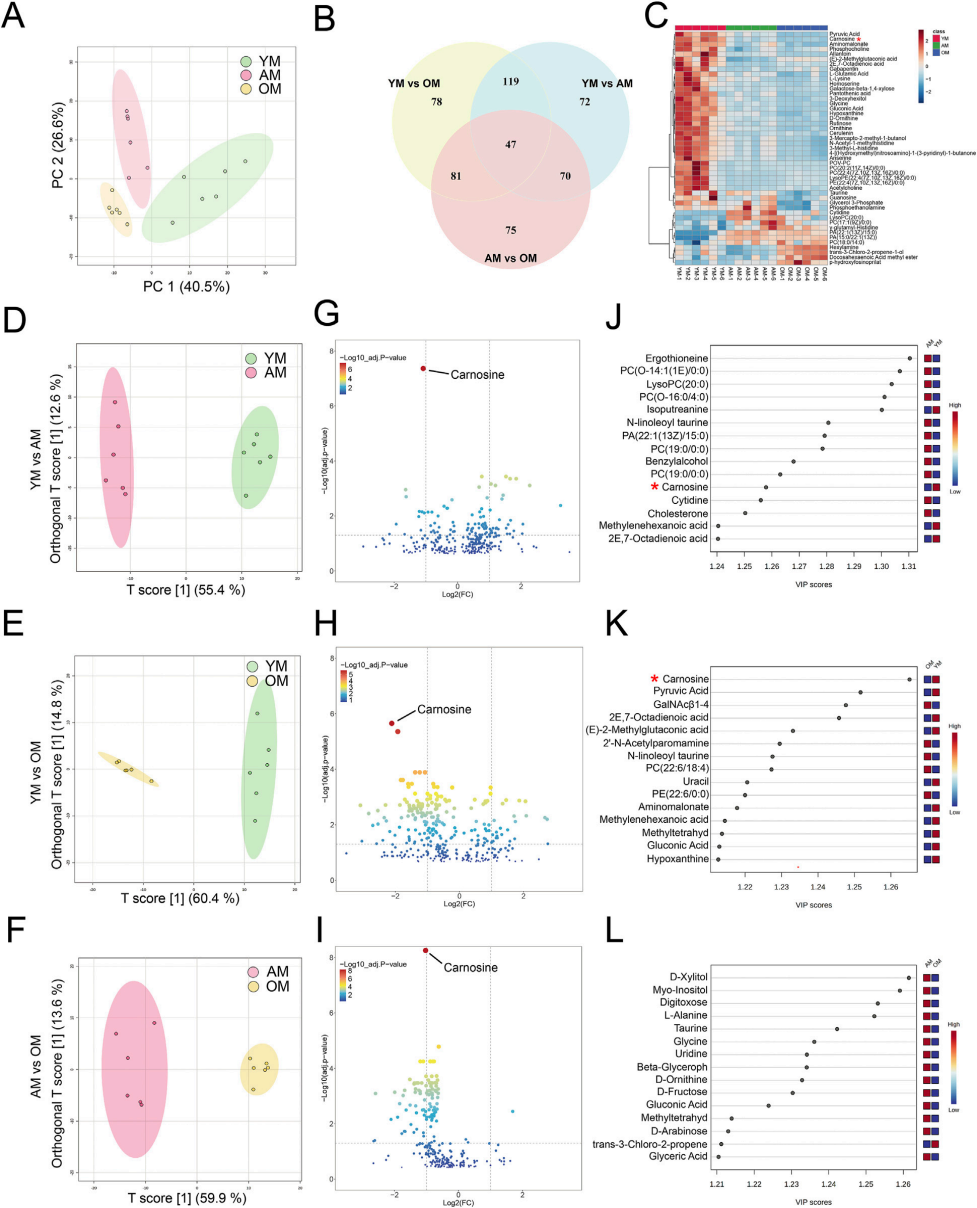
Metabolomic features associated with aging in mouse tongues. **(A)** PCA reveals significant variance in metabolites among YM, AM and OM. PC1: 40.5%, PC2: 26.6%. **(B)** A Venn plot comparing the numbers of differential metabolites showed variations between each pair of groups. **(C)** Heatmap of 47 differential metabolites overlapping in YM vs. AM, YM vs. OM and AM vs. OM. **(D–F)** Differences in metabolomic profiles between **(D)** YM and AM, **(E)** YM and OM, and **(F)** AM and OM were illustrated by the OPLS-DA score plots. **(G–I)** Volcano plots showed the alteration of metabolite concentrations between **(G)** YM and AM, **(H)** YM and OM, and **(I)** AM and OM. The points that represented carnosine were annotated. The horizontal reference dashed line indicates the adjusted *p*-value equal to 0.05. The vertical reference dashed line indicates log_2_FC values equal to −1 and 1. The size of the points is proportional to the absolute value of log_2_FC. **(J–L)** Top 15 metabolites were identified by VIP score plots, ranked by VIP values from OPLS-DA, between **(J)** YM and AM, **(K)** YM and OM, and **(L)** AM and OM.

To investigate the metabolic perturbations in oral mucosa during aging, we performed untargeted metabolomics analysis on tongues from young, adult and old mice for the first time. We finally identified 4,399 and 251 metabolite features in LC-MS and GC-MS, respectively. Following the removal of the duplicate data, the GC-MS and LC-MS data were merged, and finally, 335 differential metabolites were identified. Then we performed an unsupervised principal component analysis (PCA) to identify potential effects of age on the metabolomic profiles alteration. The PCA model showed clear separation among YM, AM and OM (Figure 2A). There were 47 metabolites with significant differences overlapping the three groups (Figure 2B). The levels of these 47 metabolites were illustrated in a heatmap (Figure 2C). Moreover, orthogonal partial least squares discriminant analysis (OPLS-DA) showed significant differences between each pair of groups, which were YM vs. AM, YM vs. OM, and AM vs. OM (Figures 2D–F). The volcano plots showed the differential metabolites with decreased expression were notably dominant, especially in the comparison of AM and OM (Figures 2G–I). Notably, carnosine consistently ranked first in all three groups, exhibiting the highest significance. Furthermore, we ranked the top 15 differential metabolites contributing to group separation according to the VIP values in each pair of groups (Figures 2J–L). We found that amino acids, peptides, and carbohydrates accounted for most of the top 15 metabolites, and carnosine, L-2-amino-3-methylenehexanoic acid, 2E,7-octadienoic acid, methyl-tetrahydrophenanthrenone, and gluconic acid were found to overlap among the three groups.

**FIGURE 3:**
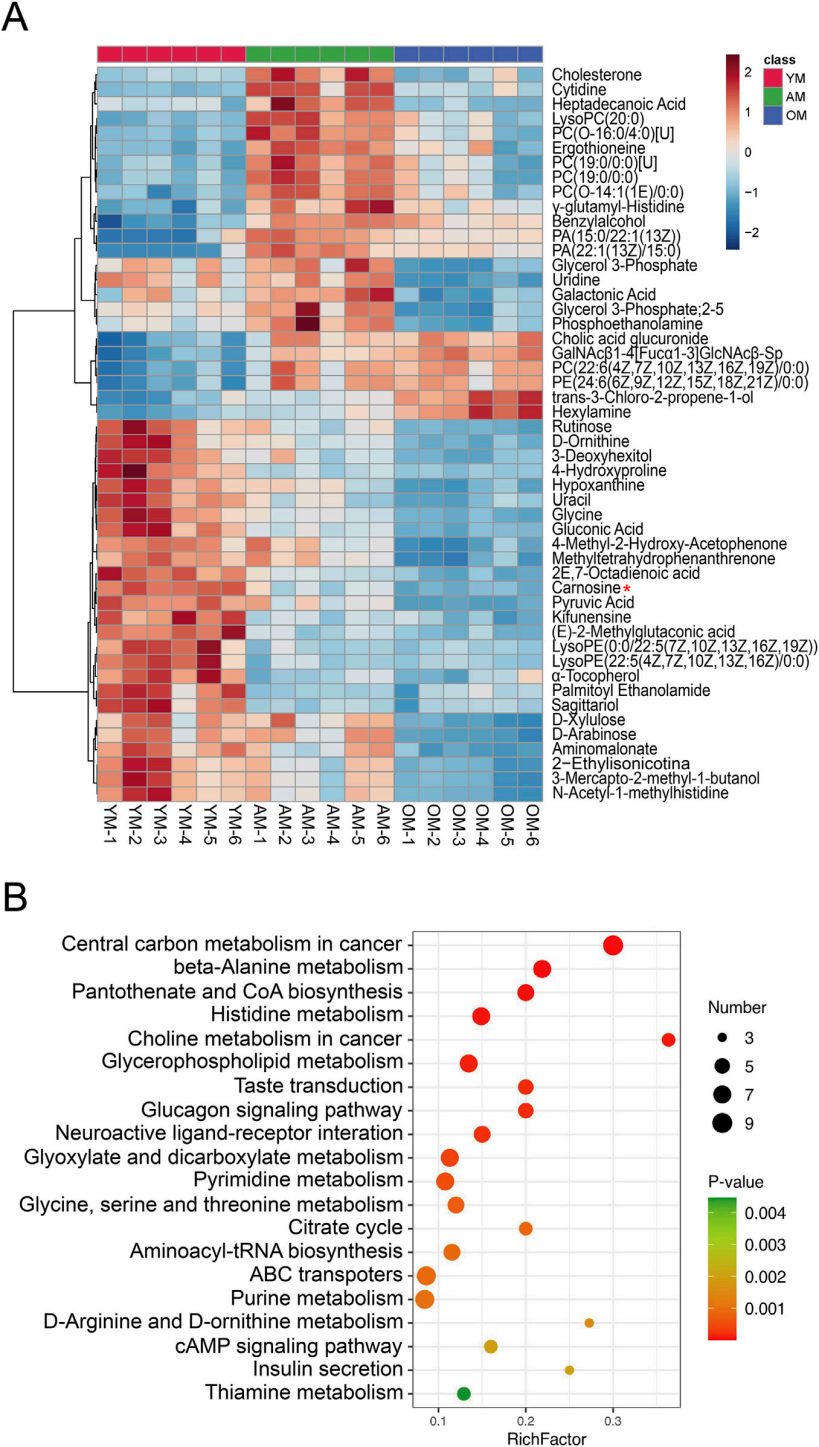
Cluster analysis and pathway enrichment analysis. **(A)** Heatmap of the top 50 significant metabolites across three different age groups, ranked by adjusted *p*-value. **(B)** Pathway enrichment analysis visualized by a bubble plot, highlighting the top 20 significant pathways. The size of the points is proportional to the number of metabolites enriched in specific pathways, while the color indicates the *p*-value. The X-axis represents the rich factor.

**FIGURE 4:**
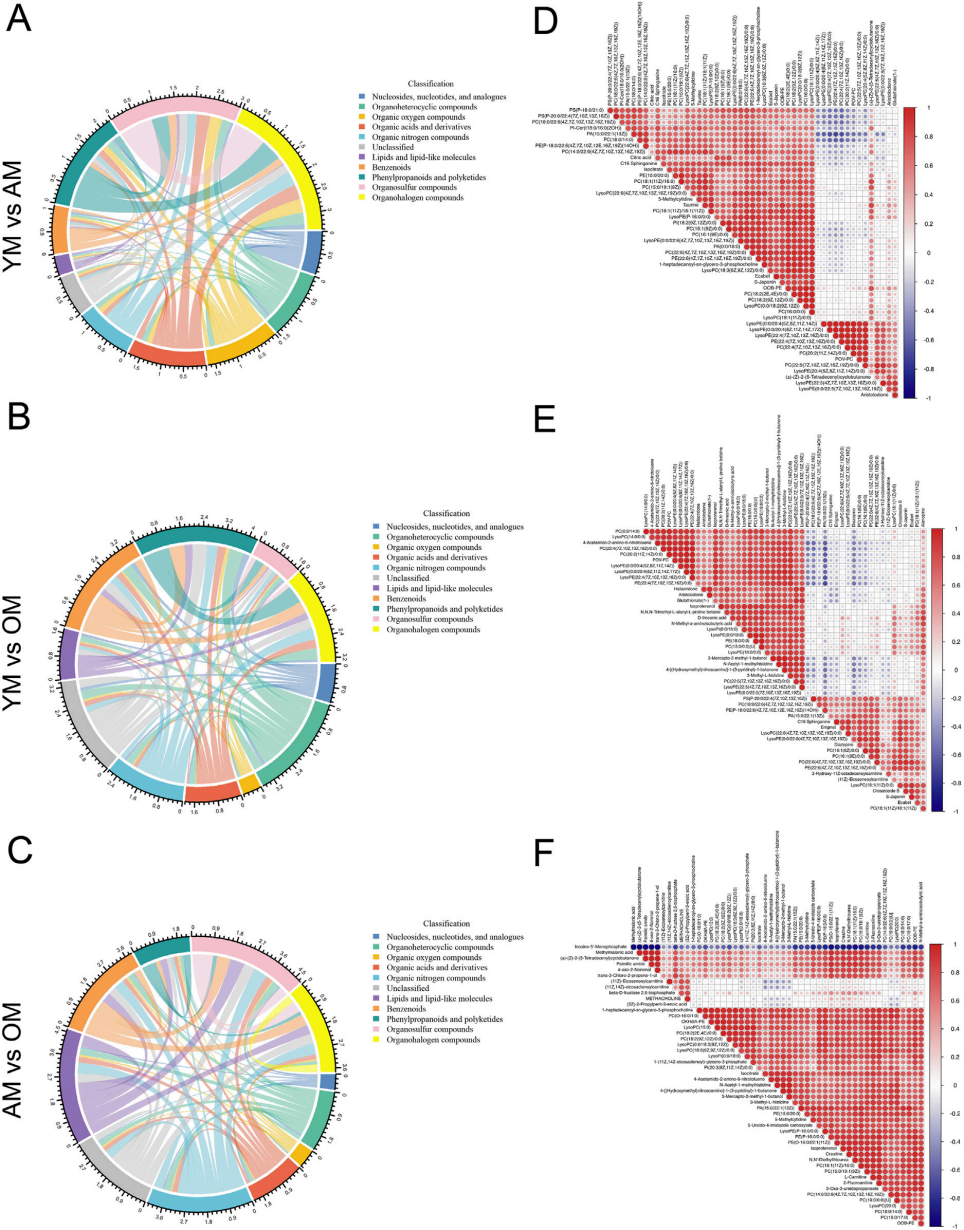
Correlation analysis of differential metabolites associated with aging. **(A–C)** The metabolic correlations between **(A)** YM and AM, **(B)** YM and OM, and **(C)** AM and OM were shown by chord diagrams. The outer ring illustrates the sum of Spearman’s correlation efficiency for each metabolic classification according to HMDB classification. The width of the bands indicates the mean correlations efficiency, and the color indicates the classification of metabolites. *P*-value cut-off was set to *p* < 0.05 and *p*-value cut-off was set to *ρ* < −0.5 or *ρ* > 0.5. **(D–F)** Correlation heatmaps show the Spearman’s rank correlation coefficients of the top 50 metabolites between **(D)** YM and AM, **(E)** YM and OM and **(F)** AM and OM. Red indicates positive correlations and blue indicates negative correlations.

Hierarchical clustering and heatmap profiling of the top 50 differential metabolites among the three paired groups (Figure 3A) demonstrated three main patterns of metabolic perturbation: a steady decrease with age, a continuous increase with age, and an increase from young to adult followed by a decrease from adult to elderly. Most of the differential metabolites decreased continuously with age. Pathway enrichment analysis highlighted the critical role of amino acid metabolism, particularly beta-alanine metabolism and histidine metabolism, both of which are linked to carnosine metabolism (Figure 3B). According to the chord diagrams, lipids and lipid-like molecules showed strong correlations across all three comparisons, indicating that these molecules may play a significant regulatory role in the aging process (Figures 4A–C). The correlation heatmap showed a high correlation between phosphocholine and lysophosphatidylethanolamine during aging (Figures 4D–F). Collectively, we found significant metabolic alterations associated with aging in the tongue mucosa of mice. During aging, the levels of most differential metabolites gradually decreased, with amino acid metabolism and carbohydrate metabolism playing important roles in determining the overall metabolic pattern.

### 3.3 Downregulation of carnosine metabolism in mouse tongue mucosa with age

**FIGURE 5:**
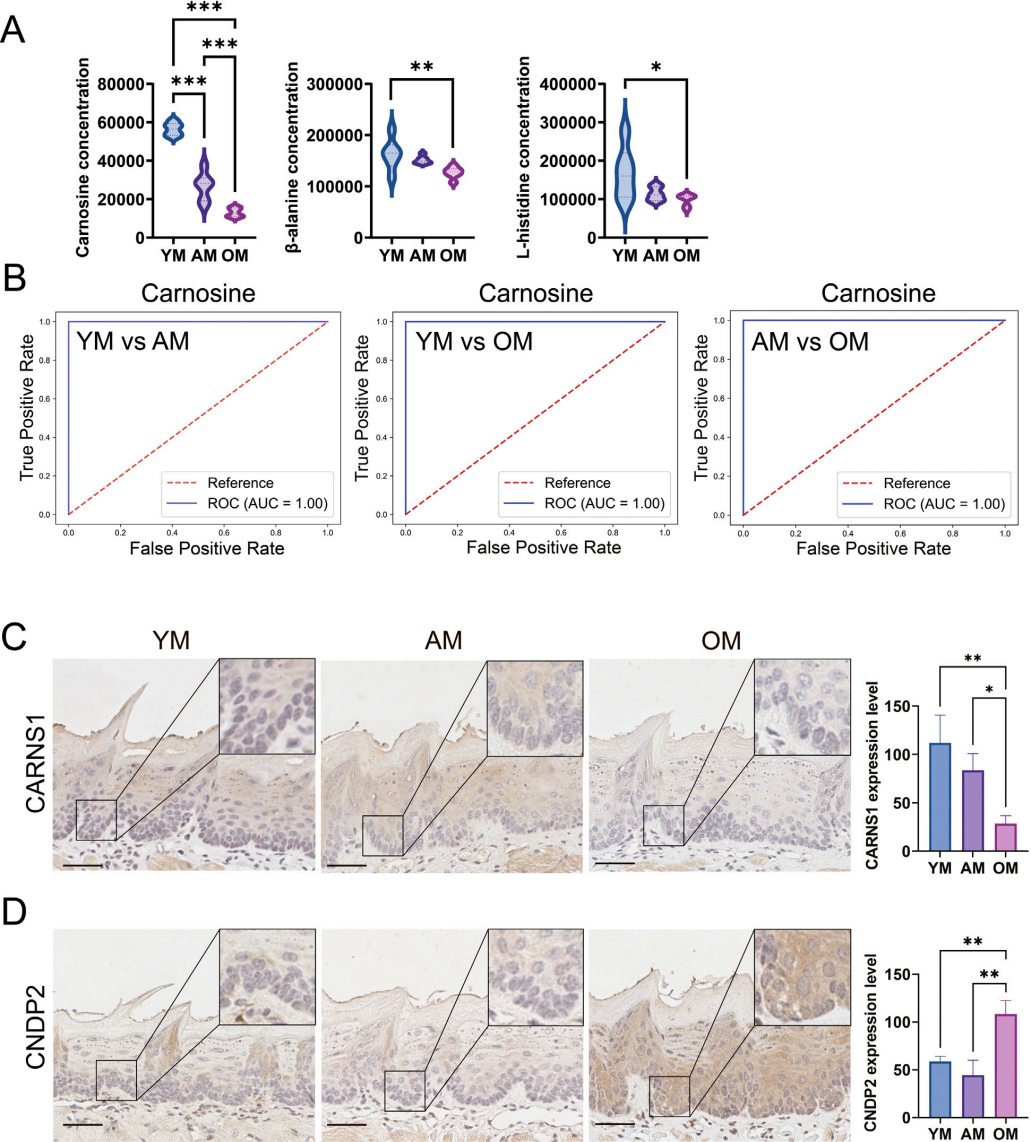
Downregulation of carnosine metabolism in mouse tongue mucosa with age. **(A)** Relative levels of carnosine, β-alanine and L-histidine in YM, AM and OM. **(B)** ROC curve analysis evaluated the accuracy of carnosine as a biomarker in distinguishing YM, AM and OM. In the YM vs. AM, YM vs. OM, and AM vs. OM groups, the AUC value was equal to 1, indicating that the predictive model based on the concentration of carnosine exhibited extremely high accuracy in distinguishing among the three groups. **(C,D)** Representative images of immunohistochemical staining and semi-quantification of **(C)** CARNS1 and **(D)** CNDP2 in tongue epithelium from young, adult and old mice. Scale bars: 50 μm (Mean ± SEM, one-way ANOVA, *p < 0.05, **p < 0.01 and ***p < 0.001, multiple correction test by Bonferroni correction).

According to the metabolomics data, the levels of carnosine, L-histidine and β-alanine in mouse tongues decreased with age (Figure 5A). Notably, the fold change of carnosine level between OM and YM is 0.23. Additionally, ROC curve analysis was performed to evaluate the potential of carnosine as a biomarker for oral mucosal aging. In the YM vs. AM, YM vs. OM, and AM vs. OM groups, the AUC value was equal to 1, indicating that the predictive model based on the concentration of carnosine exhibited extremely high accuracy in distinguishing among the three groups (Figure 5B). To further investigate the metabolic activity of carnosine, we measured the expression of CARNS1 and CNDP2 in the tongue mucosa of mice. CARNS1 expression significantly declined in OM, with no significant differences between YM and AM (Figure 5C). Conversely, CNDP2 expression increased significantly in OM, with no notable changes between YM and AM (Figure 5D). These results suggested that the age-related decline in carnosine level in the tongue mucosa might be attributed to increased hydrolysis and reduced synthesis. Overall, these findings highlight the potential of carnosine as a biomarker of tongue mucosal aging.

### 3.4 Carnosine alleviates cellular senescence induced by tBHP

**FIGURE 6:**
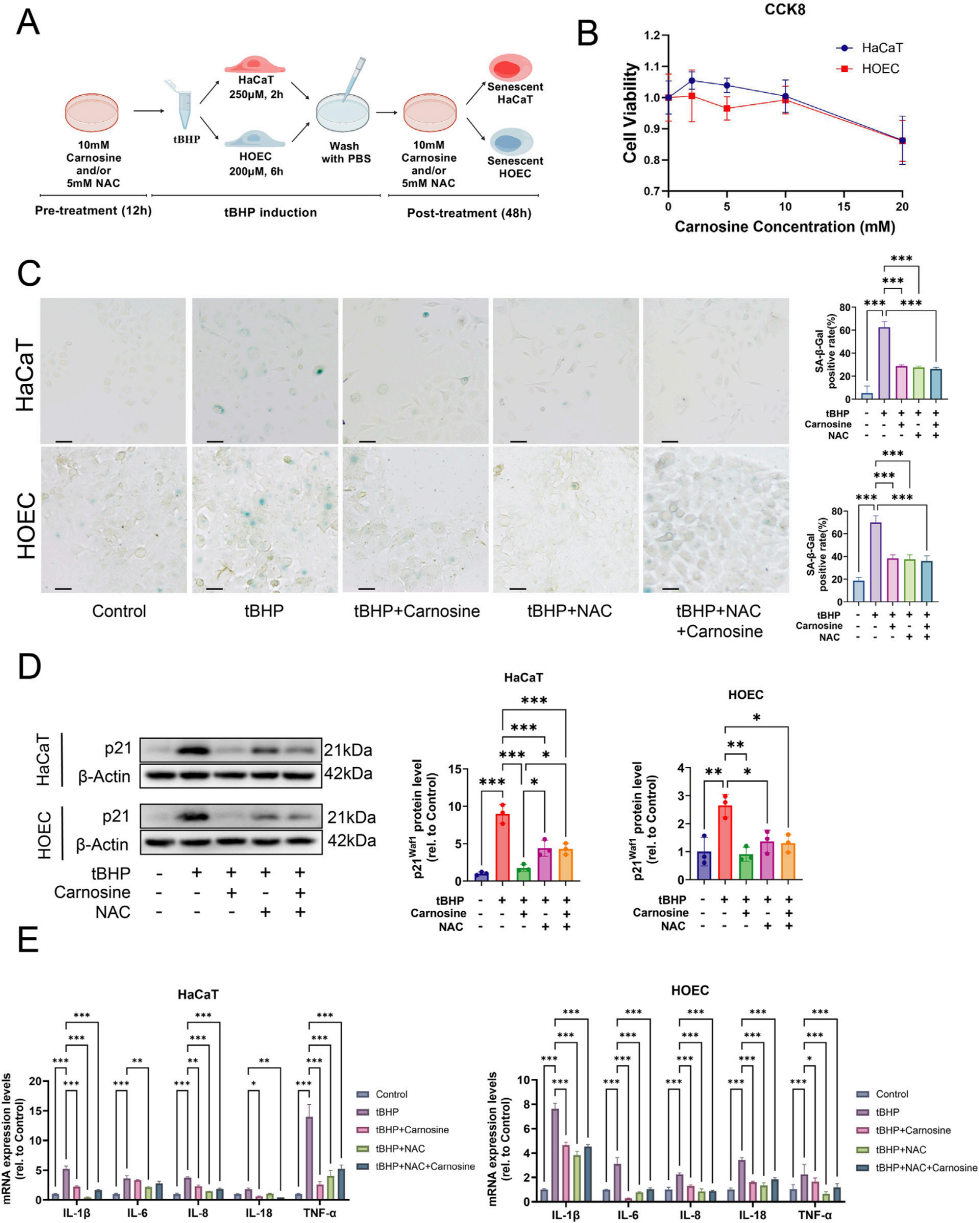
Carnosine inhibits cellular senescence *in vitro*. **(A)** Schematic diagram outlining the experimental protocol for inducing cellular senescence *in vitro*. Created with https://BioGDP.com. **(B)** Cell viability of epithelial cells after carnosine supplementation, as determined by the CCK-8 assay (n = 3). **(C)** Representative images of SA-β-Gal staining were observed under a light microscope and a quantitative analysis of the proportion of SA-β-Gal positive cells in HaCaT and HOEC was performed, respectively (n = 3). Scale bar: 100 μm. **(D)** The expression levels of p21^Waf1^ were measured by Western blot and semi-quantification in HaCaT and HOEC, respectively (n = 3). **(E)** Relative mRNA expression levels of IL-1β, IL-6, IL-8, IL-18 and TNF-α were determined by qRT-PCR in HaCaT and HOEC, respectively (n = 3) [Data represent the Mean ± SEM from n (described above) independent experiments; one-way ANOVA, *p < 0.05, **p < 0.01 and ***p < 0.001].

To investigate whether carnosine supplementation can inhibit cellular senescence, we used tBHP to establish an oxidative stress-induced cellular senescence model in HaCaT and HOEC *in vitro*, as depicted in the schematic diagram (Figure 6A). Based on the CCK-8 assay, the maximum non-toxic concentration of carnosine was determined to be 10 mM for both HaCaT and HOEC (Figure 6B). Our previous study showed the optimal NAC concentration to inhibit oxidative stress in oral epithelial cells is 5 mM (Lu et al., 2023). As hypothesized, the proportion of SA-β-Gal positive cells increased significantly after tBHP induction and decreased significantly following carnosine and/or NAC supplementation (Figure 6C). Additionally, the expression of the senescence-associated protein p21^Waf1^ increased in the tBHP group and decreased following carnosine and/or NAC intervention (Figure 6D). Notably, carnosine decreased the expression of p21^Waf1^ more effectively than NAC. Furthermore, we investigated the transcriptional activity of SASP factors, including IL-1β, IL-6, IL-8, IL-18 and TNF-α. Except for IL-6 in HaCaT, the mRNA levels of all SASP factors were significantly upregulated in tBHP-induced cellular senescence and downregulated after carnosine and/or NAC treatment (Figure 6E). These findings indicated carnosine significantly alleviated tBHP-induced cellular senescence in HaCaT and HOEC.

### 3.5 Carnosine inhibits ROS accumulation and DNA damage in tBHP-induced cellular senescence

**FIGURE 7:**
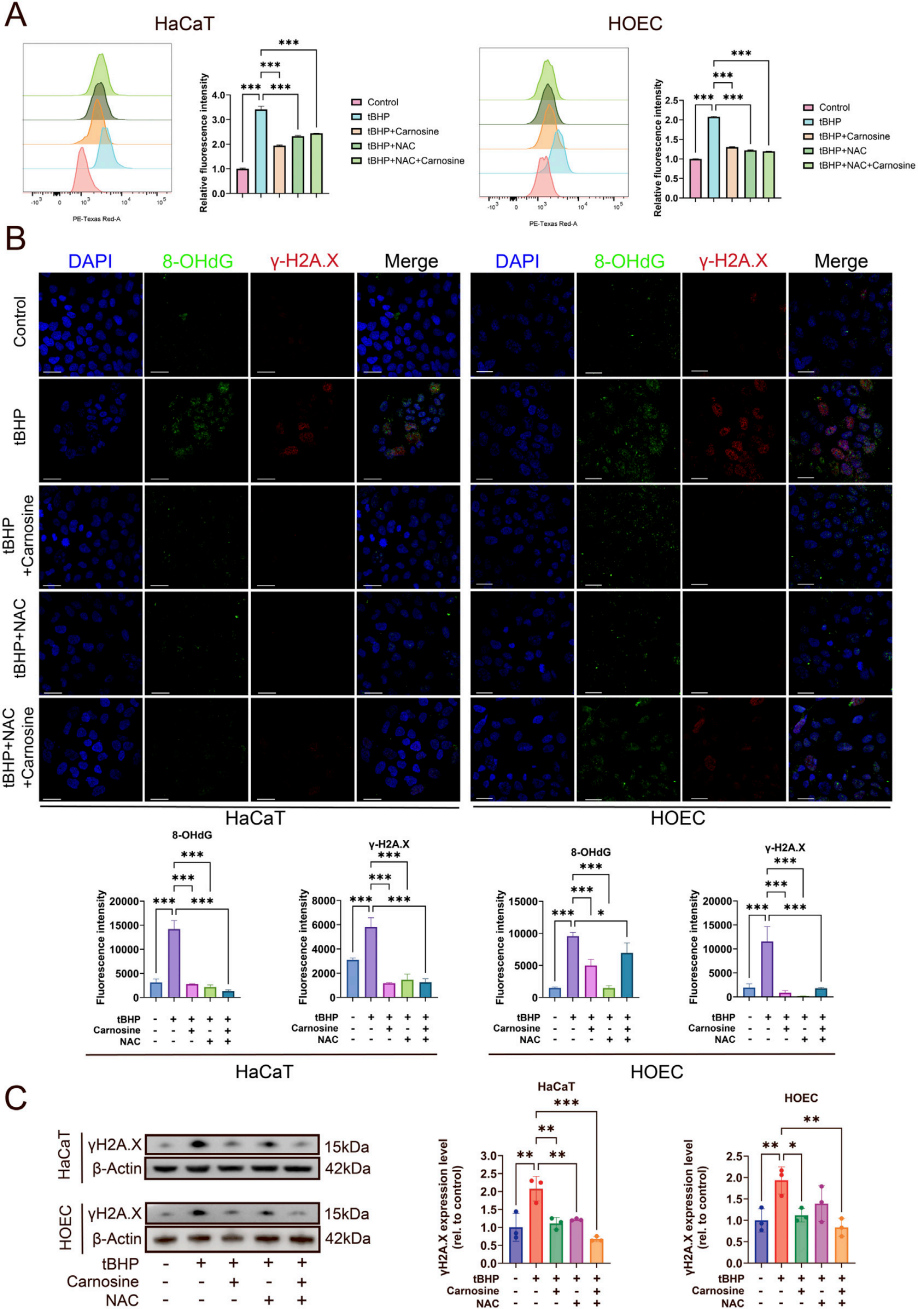
Carnosine inhibits ROS accumulation and DNA damage in tBHP-induced cellular senescence. **(A)** ROS levels were determined by flow cytometry and illustrated by histograms in HaCaT and HOEC (n = 3). ROS levels were increased significantly by tBHP induction and were relieved after carnosine and NAC intervention individually or in combination. **(B)** Representative IF images of 8-OHdG and γH2A.X in HaCaT and HOEC in each group (n = 3). Nuclei were stained with DAPI. Scale bar: 30 μm. Quantification of the mean fluorescent intensity of each group was shown below the images. **(C)** γH2A.X expression levels were determined by Western blot (n = 3) [Data represent the Mean ± SEM from n (described above) independent experiments; one-way ANOVA, *p < 0.05, **p < 0.01 and ***p < 0.001].

Firstly, the ROS levels, determined by DHE, were increased significantly after tBHP induction and were reduced significantly following carnosine and/or NAC intervention (Figure 7A). However, the combination of NAC and carnosine did not provide additional effects beyond either treatment alone in reducing ROS levels. Secondly, to further assess the DNA damage induced by ROS, we performed immunofluorescence staining of 8-OHdG and γH2A.X, which were the markers of double-strand break and DNA damage response (Figure 7B). The expression of 8-OHdG and γH2A.X was significantly increased following tBHP induction and significantly decreased after treatment with carnosine and/or NAC. Thirdly, the results of the Western blot further confirmed that the expression of γH2A.X was significantly decreased following carnosine and/or NAC intervention (Figure 7C). These findings suggest that ROS levels and oxidative stress-induced DNA damage are increased in the tBHP-induced cellular senescence and alleviated by carnosine intervention.

### 3.6 Carnosine inhibits tBHP-induced cellular senescence through downregulation of the Nrf2/HO-1 pathway

**FIGURE 8:**
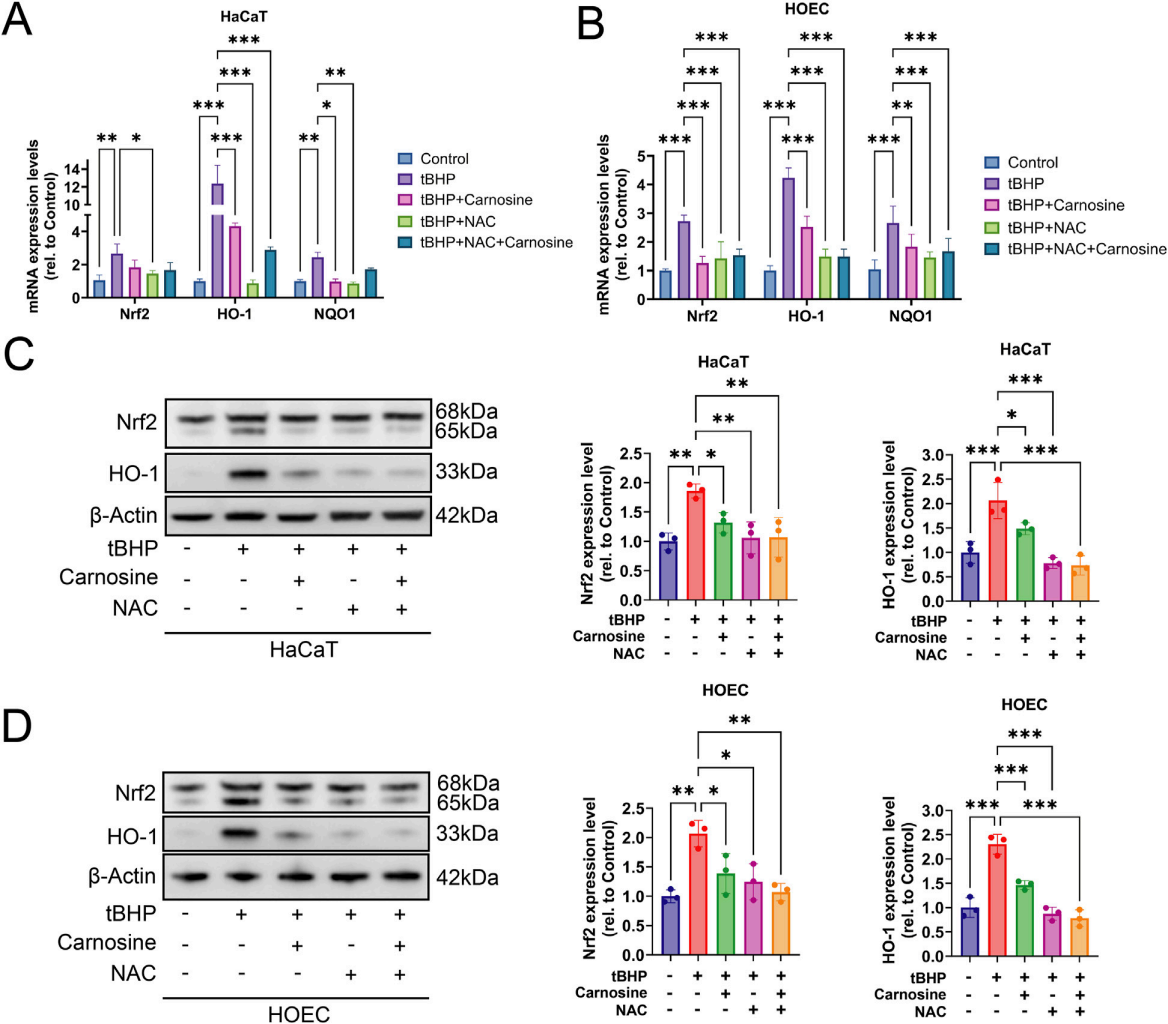
Carnosine inhibits tBHP-induced cellular senescence through downregulation of the Nrf2/HO-1 pathway. **(A,B)** mRNA expression levels of Nrf2, HO-1 and NQO1 were significantly increased in tBHP-induced cellular senescence and significantly decreased after carnosine and/or NAC intervention in HaCaT and HOEC, respectively (n = 3). **(C,D)** Protein expression levels of Nrf2 and HO-1 were decreased significantly following carnosine and/or NAC treatment, as determined by Western blot, in HaCaT and HOEC (n = 3) [Data represent the Mean ± SEM from n (described above) independent experiments; one-way ANOVA, *p < 0.05, **p < 0.01 and ***p < 0.001].

To further investigate whether the Nrf2 signaling pathway participates in the carnosine activity on cellular senescence, we evaluated the mRNA expression levels of Nrf2, HO-1 and NQO1 through qRT-PCR. As anticipated, the mRNA expression levels of Nrf2, HO-1 and NQO1 increased after tBHP induction and decreased following carnosine and/or NAC intervention (Figures 8A,B). We further confirmed the expression of Nrf2 and HO-1 at the protein level using Western blot analysis, which showed a significant increase in the tBHP-induced group and a significant decrease in the groups treated with carnosine and/or NAC (Figures 8C,D). Collectively, these results suggest that carnosine inhibits tBHP-induced cellular senescence in HaCaT and HOEC by downregulation of the Nrf2/HO-1 pathway.

### 3.7 Carnosine and its derivatives decline with age and are reduced in age-associated pathologies in human

**FIGURE 9:**
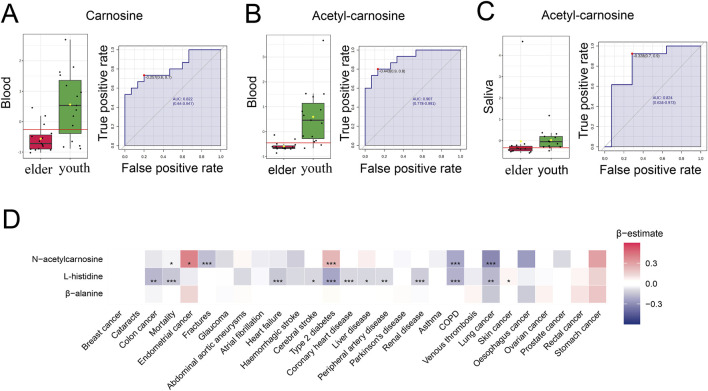
Carnosine and its derivatives decline with age and are reduced in age-associated pathologies in humans. **(A,B)** ROC curves for carnosine and acetyl-carnosine and their distribution profiles in youth and older individuals’ blood (n = 30). Both carnosine and acetyl-carnosine are decreased in elder individuals. **(C)** ROC curves for acetyl-carnosine and its distribution profile in youth and elder individuals’ saliva (n = 27). Acetyl-carnosine was decreased in elder individuals’ saliva and the AUC value was 0.824 (95% confidence interval: 0.634–0.973). **(D)** Heatmap showing the results from Cox-proportional hazard models for assessing the associations between 27 NCDs and carnosine-related metabolites (N-acetylcarnosine, histidine, and β-alanine) in blood from 11 966 subjects in the EPIC-Norfolk study. Effect size and direction of these associations are given by the β-estimates resulting from these regression models. A negative β-estimate (blue color) indicates an inverse association and a positive β-estimate (red color) indicates a positive association (Mean ± SEM, *p < 0.05, **p < 0.01 and ***p < 0.001).

Next, we sought to further investigate the relationship between carnosine and aging in the human body. Firstly, we performed ROC analysis of the metabolome in blood samples from 15 young and 15 elderly individuals (Chaleckis et al., 2016). We found the levels of carnosine and acetyl-carnosine were significantly decreased in the blood of elder individuals compared to young individuals. The AUC values for carnosine and acetyl-carnosine were 0.822 and 0.929, respectively (Figures 9A,B). Secondly, we investigated the alterations of acetyl-carnosine level in the metabolome of saliva samples from 13 young and 14 elderly individuals (Teruya et al., 2021). Acetyl-carnosine levels decreased significantly in the saliva of elderly people, with an AUC value of 0.824 (Figure 9C). Next, to explore the relationship between carnosine-related metabolites (N-acetylcarnosine, histidine, and β-alanine) and health variables in humans, we performed an association analysis between the levels of these metabolites and the risk of 27 incident non-communicable diseases (NCDs) as well as all-cause mortality in 11,966 subjects of the EPIC-Norfolk study (Figure 9D) (Pietzner et al., 2021). We found that higher levels of N-acetylcarnosine were associated with a lower prevalence of fractures, COPD and lung cancer, as well as reduced mortality. Furthermore, histidine blood level showed a negative correlation with mortality and multiple age-related diseases, such as heart failure, cerebral stroke, type 2 diabetes, coronary heart disease and COPD. Together, these findings suggest that carnosine can serve as a potential biomarker of aging, which is significantly associated with age-related pathologies in humans.

## 4 Discussion

During aging, the human oral mucosa undergoes a series of physiological and pathological alterations (Ib et al., 2016). In terms of histology, the oral mucosa in humans demonstrates decreased elastic fibers and disorganization of collagen bundles in the connective tissue (Ib et al., 2016). A recent study on geriatric cynomolgus monkeys indicated that the thickness of gingival lamina propria significantly decreased with aging, and collagen bundles significantly decreased in aged males but not in aged females (Hu et al., 2024). Previous studies have proved decreased epithelial thickness accompanied with increased thickness of the keratin layer in the mouse tongue mucosa during aging (Carrard et al., 2008). Nevertheless, in our study, we found epithelial thickness decreased significantly while the thickness of the keratin layer did not increase significantly in OM compared with YM and AM. This contrary evidence suggested that the adaptive response, which was characterized by an increased keratin layer in response to reduced epithelial thickness, was not as significant as previously thought and that further studies with larger samples were needed.

In addition, the oral mucosal epithelium contains a substantial reservoir of epithelial stem cells essential for maintaining epithelial homeostasis (Papagerakis et al., 2014). The basal cells of oral mucosal epithelium become larger and flatter with aging, accompanied by a decreased cell proliferation rate (Carrard et al., 2008). In our study, we observed an accumulation of senescent cells in tongue epithelium, as indicated by a higher positive rate of SA-β-Gal and p21^Waf1^ in elderly mice compared with young and adult mice. DNA damage, a well-established hallmark of aging (López-Otín et al., 2023), was also found to be increased in the tongue epithelium of elderly mice, evidenced by increased levels of 8-OHdG in comparison to the younger mice. Moreover, the reduced proliferation rate was supported by a decreased Ki-67 positive rate in epithelial cells during aging. Functional decline is another characteristic of oral mucosal aging. For instance, gingival wound healing is severely delayed with aging (Smith et al., 2015). Our findings suggest that the accumulation of senescent cells in epithelium may contribute to the impaired wound healing in oral mucosa during aging.

To find out the age-associated metabolic alterations *in situ*, we comprehensively analyzed the metabolic characteristics of aging in mouse tongues for the first time and identified a total of 4,650 metabolite features using a combination of LC-MS and GC-MS methods. Our findings provide specific insights into age-related metabolic changes in mouse tongues, suggesting that aging has a profound effect on the metabolic characteristics of this tissue. Most of the differential metabolites decreased in OM compared with YM and AM. According to the results of pathway enrichment analysis and correlation analysis, alterations in amino acid metabolism and carbohydrate metabolism play a pivotal role in aging in mouse tongues. We also identified carnosine as a potential biomarker of oral mucosal aging. Carnosine is a naturally occurring dipeptide that has been used as an antioxidant and anti-glycating agent to protect against age-related disorders and facilitate healthy aging (Turner et al., 2021). A recent study indicated that carnosine stimulated macrophage-mediated clearance of senescent skin cells, though it did not directly eliminate senescent cells after tBHP induction (Li et al., 2020). However, our results demonstrated that carnosine supplementation significantly protected against tBHP-induced cellular senescence in HaCaT and HOEC. These findings suggest that carnosine protected cells from cellular senescence through blocking senescence induction rather than clearing senescent cells.

Carnosine not only directly scavenges free radicals but also indirectly enhances endogenous antioxidant systems, chelates metal ions, and inhibits protein carbonylation and glycoxidation (Caruso et al., 2023). The primary antioxidant capacity of carnosine is attributed to the imidazole ring of its L-histidine residue, which reacts directly with ROS to diminish the oxidative reactivity of free radicals. Notably, carnosine has been reported to react with singlet oxygen two-to four-fold faster than free L-histidine (Boldyrev et al., 2013). In this study, carnosine significantly reduced ROS levels in senescent cells. This finding was further supported by Western blot and qRT-PCR results, which showed that carnosine markedly decreased the activation of Nrf2 and its downstream molecules in senescent cells. These results suggest that carnosine inhibits cellular senescence by directly scavenging ROS rather than by indirectly activating endogenous antioxidant systems. Moreover, carnosine is an effective quencher of hydroxyl radicals (^·^OH), which is a primary cause of DNA damage. DNA damage triggers a DNA damage response and subsequently activates downstream signaling cascades involving p53/p16 and p21/Rb that lead to cell cycle arrest (Shmulevich and Krizhanovsky, 2021). Our results of immunofluorescence and Western blot analysis showed that both carnosine and NAC significantly reduced the expression of DNA damage markers, including 8-OHdG and γH2A.X, in senescent cells. Although the effects of carnosine on ROS scavenging and DNA protection were comparable to those of NAC, carnosine more effectively downregulated the expression of senescence markers such as p21^Waf1^. These results suggest that carnosine is more effective than NAC at inhibiting cellular senescence, likely due to its additional ability to inhibit protein carbonylation and glycoxidation, thereby preserving protein structure and function (Hipkiss et al., 2016).

Nrf2 is a critical transcription factor that regulates more than 600 genes involved in responses to oxidative stress (Wang et al., 2022). It plays a protective role in inhibiting age-related pathologies caused by oxidative damage and inflammation, such as Alzheimer’s disease, Parkinson’s disease, Huntington’s disease, amyotrophic lateral sclerosis, stroke, and multiple sclerosis (George et al., 2022). Nrf2 has been shown to protect against oral mucositis via antioxidation and keratin layer thickening (Wakamori et al., 2022). While Nrf2 activation is generally linked to enhanced defense against oxidative stress, our study showed that tBHP induction increased Nrf2 and HO-1 expression. After carnosine and/or NAC intervention, the expression levels of both Nrf2 and HO-1 were significantly reduced, indicating that carnosine and/or NAC alleviated oxidative stress. Our findings were in line with earlier research on oxidative stress-induced skin damage (Wang et al., 2020). These findings indicated that carnosine downregulated the Nrf2 signaling pathway in keratinocytes to inhibit oxidative stress-induced cellular senescence. It is also important to note that prolonged activation of Nrf2 has negative effects. Long-term activation of Nrf2 in keratinocytes can result in epidermal thickening, hyperkeratosis and inflammation in mice (Schäfer et al., 2012). Activation of Nrf2 in fibroblasts induces cellular senescence and the expression of genes characteristic for cancer-associated fibroblasts (Hiebert et al., 2018). These findings suggested that preventing aberrant Nrf2 pathway activity in aging may be beneficial. To sum up, the mechanisms of Nrf2 under aging and cellular senescence are not fully understood and require further study.

In our study, we further investigated the levels of carnosine-related metabolites in the blood and saliva during human aging. We found that carnosine and acetyl-carnosine significantly decreased with aging. The following ROC analysis proved the high predictive accuracy of these two metabolites as a biomarker of aging. Recent studies have shown that carnosine supplementation is beneficial to several age-related disorders, such as Alzheimer’s disease, dementia, Parkinson’s disease and type 2 diabetes (Attanasio et al., 2013; de Courten et al., 2016; Caruso et al., 2021; Hariharan et al., 2024). We found that higher N-acetylcarnosine and histidine levels in blood were associated with lower prevalence of some age-related diseases, such as COPD, lung cancer, fractures and heart failure. This evidence suggests that carnosine has the potential to serve as a biomarker of aging, both in the oral epithelium and systemically.

Carnosine was well tolerated and exhibited no toxicity. In rat models, intravenous administration of carnosine at doses ranging from 100 to 2,000 mg/kg did not result in any adverse effects. Parameters such as body weight, food consumption, activity, and mortality did not differ significantly from those in the control group, and no organ lesions were observed (Bae et al., 2013). Moreover, no severe or systemic adverse events were attributed to the subcutaneous injection of a hyaluronic acid containing L-carnosine (2.00 mg/mL) for the treatment of neck wrinkles in humans, and no allergic reactions were reported (Yue and Ju, 2022). Furthermore, Polaprezinc (PZN), a mucosal protective zinc L-carnosine complex used to treat *Helicobacter pylori* infections without the risk of resistance in humans, has also demonstrated an excellent safety profile. The typical PZN dose is 150 mg, which contains 34 mg of zinc and 116 mg of L-carnosine, and no significant adverse events were observed following its oral administration (Mahmoud et al., 2022). PZN lozenge (75 mg/day for 35 days) is effective for prophylaxis against oral mucositis associated with chemotherapy without any side effects (Cesak et al., 2023). Based on the above evidence, carnosine demonstrates high safety and tolerability and can be utilized for the treatment of oral mucosal diseases. In the future, carnosine appears to be a promising and safe therapeutic agent for addressing oral mucosal aging and aging-related disorders.

A limitation of this study is the lack of supplementation experiments to directly verify carnosine’s effect on oral mucosal aging in mouse tongues. Future studies should address this through animal models. In conclusion, our study reveals dynamic metabolic reprogramming during natural oral mucosal aging, thereby establishing carnosine’s dual role as both an aging biomarker and a therapeutic target for combating age-related mucosal degeneration. Our work not only elucidates the specific mechanism underlying the anti-senescence effect of carnosine, which involves alleviating oxidative stress, reducing DNA damage and downregulating Nrf2/HO-1 pathway, but also provides critical insight into how carnosine may function as an anti-aging pharmaceutical agent. Ultimately, our work lays the foundation for the development of innovative carnosine-based strategies aimed at preserving oral mucosal function, preventing age-related oral diseases, and enhancing oral health in the aging population. In doing so, it contributes to the broader goal of healthy aging.

## Data Availability

The raw data supporting the conclusions of this article will be made available by the authors, without undue reservation.
